# Giant parathyroid adenoma: a case report

**DOI:** 10.1186/s13256-022-03401-y

**Published:** 2022-04-13

**Authors:** Rahim Mahmodlou, Amin Sedokani, Apameh Pezeshk, Bita Najafinejad

**Affiliations:** 1 Department of Surgery, Urmia University of Medical Sciences, Urmia, Iran; 2 Department of Cardiology, Urmia University of Medical Sciences, 17 Shahrivar St., 571478334 Urmia, Iran; 3 Department of Medicine, Section of Hematology/Oncology, University of Chicago Biological Sciences, Chicago, IL USA

**Keywords:** Giant parathyroid adenoma, Parathormone, Hypercalcemia, Bone pain

## Abstract

**Background:**

Giant parathyroid adenoma is a type of parathyroid adenoma weighing > 3.5 g and having a size of more than 2 cm.

**Case presentation:**

This report describes giant primary parathyroid adenoma with reference to the literature. We report the case of a 48-year-old Persian man referred to the clinic with knee and lower back pain. He had a history of mitral valve replacement and several episodes of bilateral nephrolithiasis. After a thorough assessment, a neck mass with a possible thyroid origin was detected, but further assessment showed it was of parathyroid origin. The resected mass was 9 × 6× 4 cm and weighed 122 g, and histopathology showed a giant parathyroid adenoma.

**Conclusion:**

Giant parathyroid adenomas that weigh more than 110 g and are larger than 8 cm can lead to significant hypercalcemia. Despite giant parathyroid adenomas and high parathyroid hormone levels, a calcium crisis may not always occur in these patients, and the masses may be initially misdiagnosed as a thyroid mass.

## Background

Primary hyperparathyroidism (PHPT) is the most common disease of parathyroid glands and the third most common endocrine disease [1, 2]. The prevalence of PHPT in the general population is 22 per 100,000. In postmenopausal women, the prevalence reaches up to 1 per 500. Parathyroid adenoma is the cause of 80–90% of PHPT. The remaining 10–20% of PHPT cases are due to parathyroid hyperplasia, and < 1% are due to parathyroid carcinoma 4. Giant parathyroid adenoma (GPA) is an adenoma with a weight > 3.5 g and diameter > 2 cm. The average weight of the parathyroid gland is about 70 mg to 1 g [1, 3]. The mainstay of the pathophysiology of hyperparathyroidism is hypersecretion of parathyroid hormone (PTH), which leads to the release of calcium from bone cells by inhibiting osteoblasts and stimulating osteoclast activity. PTH stimulates calcium reabsorption and inhibits phosphate reabsorption in kidneys. Additionally, PTH stimulates calcium absorption from the gut [3] by stimulating the conversion of 25-hydroxyvitamin D to 1,25-hydroxyvitamin D.

Clinical manifestations of PHPT include nephrolithiasis, osteoporosis–osteopenia, pancreatitis, depression, cognitive disorders, and others. The severity of symptoms correlates with the adenoma’s weight and PTH level [1, 3]. Patients with hypercalcemia may experience a life-threatening hypercalcemic crisis due to the high level of PTH secondary to a parathyroid carcinoma or a giant parathyroid adenoma [4, 5]. Since the most common cause of PHPT is adenoma and most adenomas are asymptomatic, PHPT cases are mostly diagnosed during screening tests with elevated calcium and parathormone levels [2, 3].

Previously, the largest reported GPA was 8 × 5 × 3.5 cm with a weight of 110 g [6] and was associated with hypercalcemia (3.21 mmol/L or 12.84 mg/dL).

## Case presentation

A 48-year-old Persian man from Urmia, Iran, presented to Imam Hospital with knee pain, lower back pain, fatigue, and dizziness for the last 2 months. The patient had a history of mitral valve replacement 30 years earlier, several bilateral nephrolithotripsies, and diabetes mellitus under control with oral medication. His vital signs were typical. There was a palpable large and soft nodule in the left lobe of his thyroid, which moved with swallowing. A scar of previous cardiac surgery was visible on the chest. The rest of the examination was normal.

**Fig 1:**
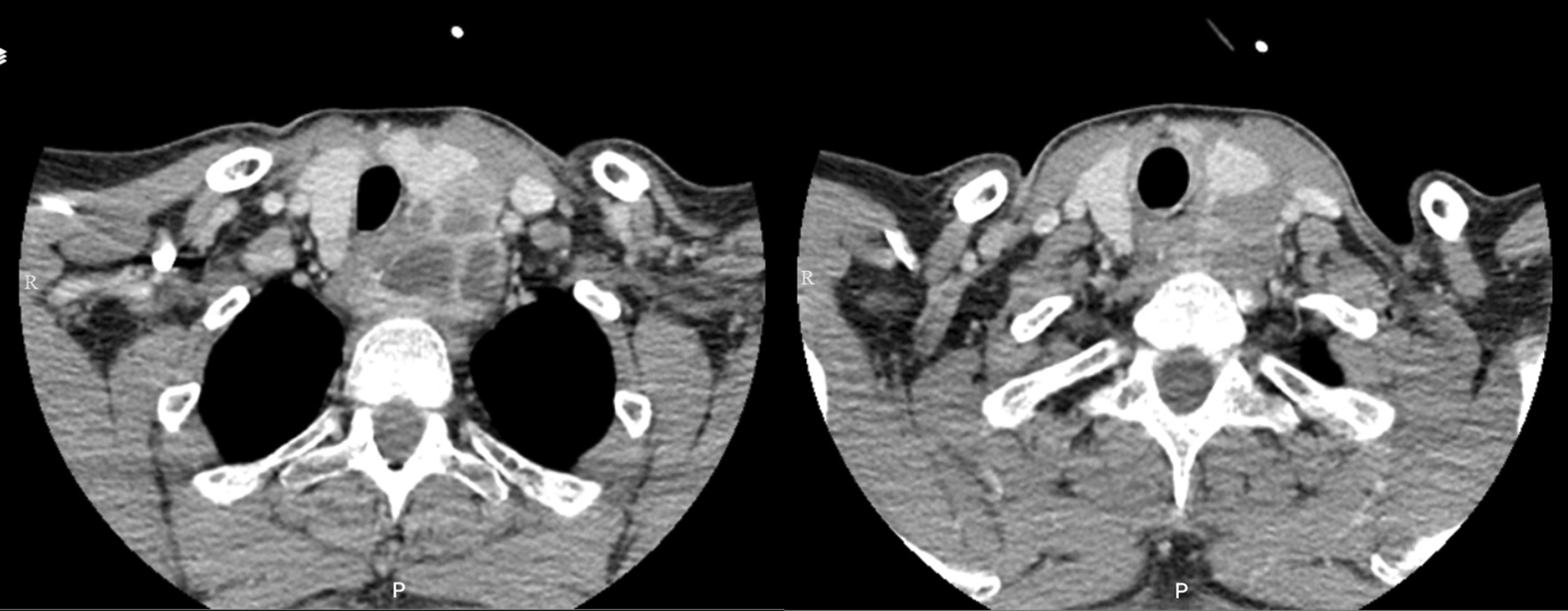
Computed tomography scans of the lower neck (right) and upper mediastinum (left) showing a vast mass pushing the trachea out of the midline

Color Doppler ultrasonography of the thyroid and parathyroid indicated a single isoechoic nodule in the right lobe (12 × 9.5 mm) and two cystic nodules in the left lobe (40 × 23 mm and 30 × 16 mm) of his thyroid. A cystic mass in the region of the left lobe of the thyroid was visible on computed tomography (CT), in contrast to the patient’s biochemistry, indicative of parathyroid carcinoma (Fig. 1). The mentioned findings were approved using ^99m^Tc-MIBI scintigraphy. His complete blood count (CBC) was normal, and the results of the biochemical tests are presented in Table 1. Calcium and PTH serum levels were 14.6 mg/dL and 2702 pg/mL, respectively. Moreover, urine analysis revealed trace proteinuria, with two to three red blood cells (RBCs) per high-power field (HPF).

**Table 1:** Biochemical and urine analysis tests of the patient

Variable (serum)	Patient’s values	Reference range	Unit
Calcium	14.6	8.5–10.5	mg/dL
Sodium (Na)	138	136–145	mEq/L
Potassium (K)	4.3	3.5–5.5	mEq/L
Magnesium (Mg)	2.04	1.8–2.6	mg/dL
Phosphorous (P)	2.14	2.8–4.5	mg/dL
Parathyroid hormone (PTH)	2702	14–65	pg/mL
Macroscopic U/A			
Color	Yellow	Yellow	–
Appearance	Clear	Clear	–
PH	7	4.6–8	–
Protein	Trace	Negative	–
Glucose	Negative	Negative	–
Blood/Hb	Trace	Negative	–
Microscopic (U/A)			
WBC	1–2	0–2	Per HPF
RBC	4–5	0–2	Per HPF
Epithelial	2–3	0–1	Per HPF
Mucus	Negative	Negative	Per HPF

Based on the patient’s lab and imaging findings, the probability of a parathyroid carcinoma was high enough to send the patient to surgery for tumor resection.

The operation was performed under general anesthesia. After creating a large collar incision (12 cm length) two-finger width above the suprasternal notch, subplatysmal flaps were created as a routine thyroidectomy procedure. The raphe between strap muscles was opened, and the muscles were incised on the left near the upper pole of the thyroid.

**Fig 2:**
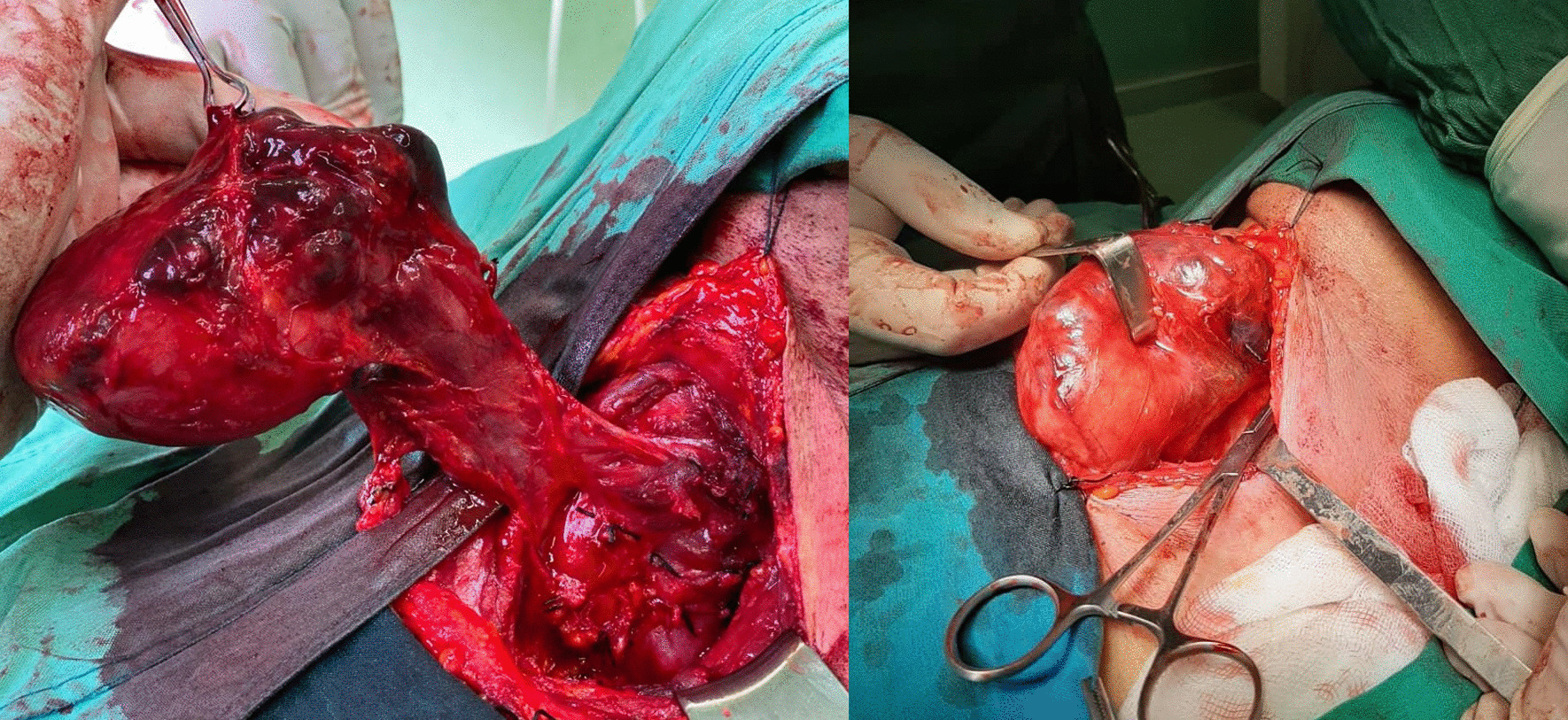
Surgical view of the mass obscuring the thyroid gland (right) and dissection (left)

On initial evaluation after the thyroid was exposed, it appeared as a thyroid mass displacing the carotid sheath laterally and extending into the mediastinum as a retrosternal goiter. However, further evaluation showed a central posterior neck mass with extension to mediastinum that displaced carotid sheath laterally and the left lobe of the thyroid superior and medially. The inferior thyroidal artery crossed the mass toward the carotid sheath. These findings favored a parathyroid origin, most likely from the superior parathyroid glands. The inferior thyroid artery was ligated as laterally as possible, and then the lateral side of the tumor was dissected out with sharp and blunt dissection, and the tumor was pulled out of the mediastinum to the neck (Fig. 2). The recurrent laryngeal nerve was explored between the trachea and medial side of the tumor, that is, the mass was located dorsal to the recurrent laryngeal nerve (further evidence of superior parathyroid origin of the tumor).

Owing to the PTH value, size of the tumor, and adhesion to adjacent tissues, the patient was diagnosed with parathyroid carcinoma, and the tumor, right lobe of the thyroid, and adjacent lymph nodes were resected. After inserting a Hemovac drain, the neck wound was closed. The resected mass had a size of 9 × 6 × 4 cm and weighed 122 g. Since the mass was descended to the retro-esophageal space and was located posterior to the recurrent laryngeal nerve, we believe it originated from the superior parathyroid gland.

The postoperative period was eventless. In the first 24 hours, an intravenous infusion (drip) of calcium gluconate was given to prevent bone hunger syndrome. Liquid diet was started in the second 24 hours, and the patient was discharged on the third postoperative day with oral CaCO_3_ and calcitriol pearls gradually tapered until discontinued after 2 weeks.

**Fig 3:**
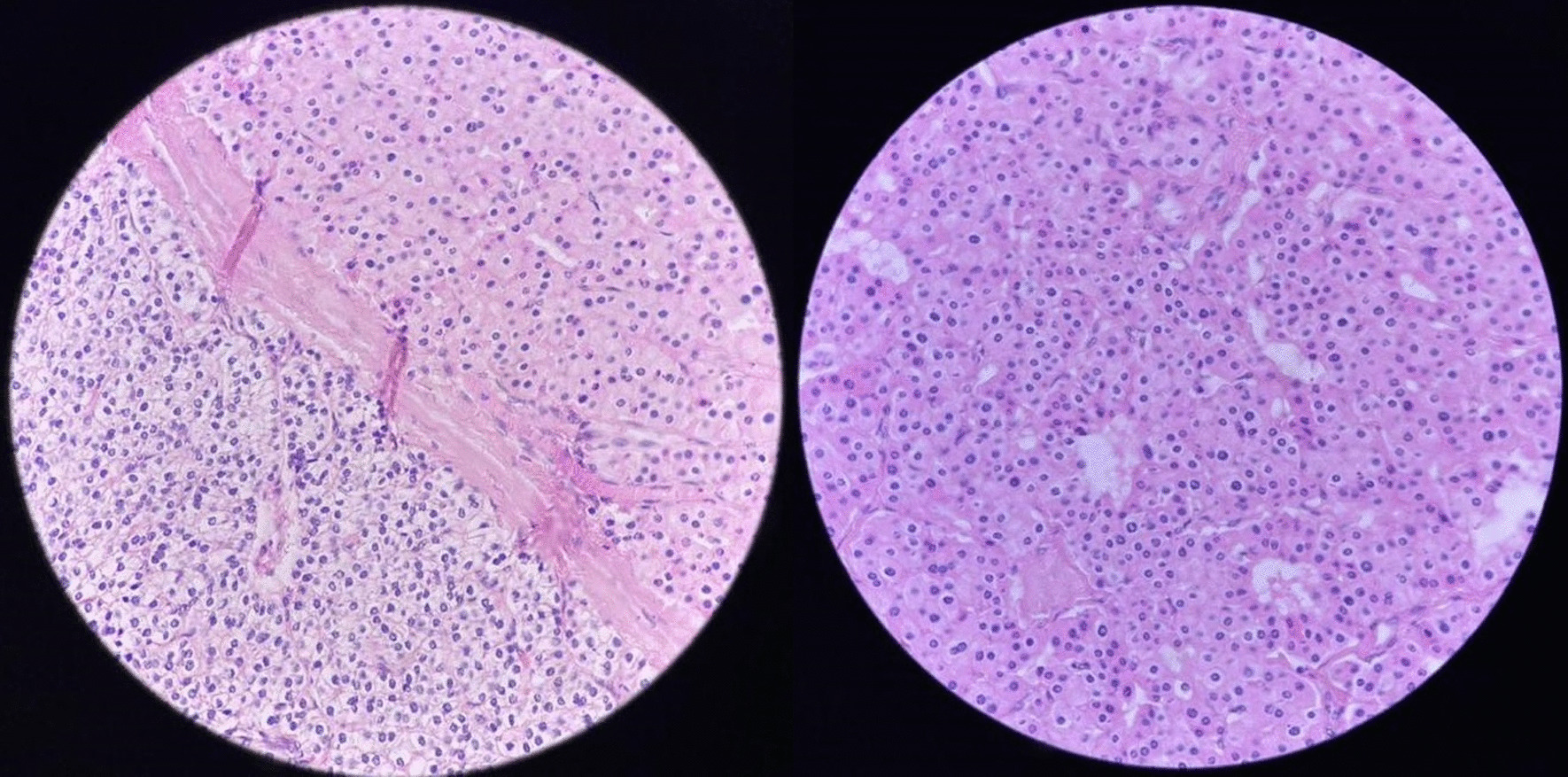
Histopathological view of the resected mass in favor of parathyroid adenoma

Histopathological assessment of the resected mass revealed a parathyroid adenoma and multinodular goiter of the resected thyroid lobe (Fig. 3). No lymph node or local invasion, vascular invasion, or perineural invasion was reported on histopathological assessment, and there was no significant invasion of adjacent structures or organs. Finally, the patient was followed up for 12 months without any problems.

## Discussion

Primary hyperparathyroidism (PHP) is the third most common endocrine disorder and is usually due to parathyroid adenoma. Parathyroid carcinoma is the least common cause of PHP but should be considered in the differential diagnosis of PHP when the serum calcium (mg/dL) level is above 14 or PHP is associated with a palpable neck mass [7–9]. The symptoms result from high levels of PTH and higher reabsorption of Ca from bones, renal tubules, and the gut, causing hypercalcemia [1, 2, 10, 11]. However, giant parathyroid adenoma can cause anatomical symptoms due to the large tumor size, including dysphagia, odynophagia, and dyspnea [12, 13]. Clinical studies show that hypercalcemia is very common in cases presenting with parathyroid mass. Owing to the increase in PTH levels and parathyroid function, ultrasonography and ^99m^Tc-MIBI scintigraphy facilitate the diagnosis of giant parathyroid adenoma [14].

The patient had been suffering from recurrent bilateral nephrolithiasis. Considering his back and bone pain, the adenoma might have been chronically functional, so the patient had not experienced calcium crisis symptoms. Diagnosis of a parathyroid mass was made on the basis of lab findings, including high calcium and PTH levels. While the CBC was as expected, the level of PTH was almost 50 times its expected value. Previous studies have shown that parathyroid carcinoma occurs with equal frequency in both sexes and usually increases serum PTH to five- to tenfold of the normal upper limit [8, 9, 11]. Furthermore, some criteria increase parathyroid carcinoma likelihood, including “local invasion of contiguous structures” or “lymph node or distant metastases.”

However, the calcium level was not as high as the PTH level, which could be due to chronic elevation of PTH or genetic resistance to PTH [15].

At that point, we had no evidence to prove either option. After tumor resection and putting the patient on calcium and calcitriol for several months, then tapering them, the patient no longer had symptoms of hypo- or hyperparathyroidism, which ruled out any genetic disorder of Ca metabolism.

In summary, hyperparathyroidism should be considered in the differential diagnosis of any neck and cervical mass, even though it is a sporadic tumor.

## Conclusion

This case report describes the largest giant parathyroid adenoma that has ever been diagnosed, although the size of the mass, location, and imaging might be misleading. It should be noted that very high levels of parathormone may not always lead to calcium crisis signs and symptoms.

## Data Availability

Not applicable.

## Abbreviations

PHPT: Primary hyperparathyroidism
GPA: Giant parathyroid adenoma
PTH: Parathyroid hormone
